# Orchestrating segment anything models to accelerate segmentation annotation on agricultural image datasets

**DOI:** 10.3389/frai.2025.1748468

**Published:** 2026-01-22

**Authors:** Leon H. Oehme, Jonas Boysen, Zhangkai Wu, Anthony Stein, Joachim Müller

**Affiliations:** 1 Institute of Agricultural Engineering, Tropics and Subtropics Group, University of Hohenheim, Stuttgart, Germany; 2 Institute of Agricultural Engineering, Department of Artificial Intelligence in Agricultural Engineering, University of Hohenheim, Stuttgart, Germany

**Keywords:** agriculture, annotation, deep learning, phenotyping, segment anything model 2, segmentation, UAV

## Abstract

Increasingly many applications of machine vision and artificial intelligence (AI) can be observed in agriculture. Yet, high-quality training data remains a bottleneck in the development of many AI solutions, particularly for image segmentation. Therefore, ARAMSAM (agricultural rapid annotation module based on segment anything models) was developed, a user interface that orchestrates the pre-labelling capabilities of both the segment anything models (SAM 1, SAM 2) and conventional annotation tools. One *in silico* experiment on zero-shot performance of SAM 1 and SAM 2 on three unseen agricultural datasets and another experiment on hyperparameter optimization of the automatic mask generators (AMG) were conducted. In a user experiment, 14 agricultural experts applied ARAMSAM to quantify the reduction of annotation times. SAM 2 benefited greatly from hyperparameter optimization of its AMG. Based on ground-truth masks matched with predicted masks, the *F_2_*-score of SAM 2 improved from 0.05 to 0.74, while that of SAM 1 was improved from 0.87 to 0.93. The user interaction time could be reduced to 2.1 s/mask on single images (SAM 1) and to 1.6 s/mask on image sequences (SAM 2) compared to polygon drawing (9.7 s/mask). This study demonstrates the potential of segment anything models as incorporated into ARAMSAM to significantly accelerate the process of segmentation mask annotation in agriculture and other fields. ARAMSAM will be released as open-source software (AGPL-3.0 license) at https://github.com/DerOehmer/ARAMSAM.

## 1 Introduction

In recent years, the rapid development of machine vision based on artificial intelligence (AI) has gained increasing attention in agriculture (Abbasi et al., 2022; Maraveas, 2024). This becomes especially apparent in the field of plant phenotyping, where AI enables more precise and efficient analysis of plant traits (Farooq et al., 2024; Sheikh et al., 2024; Visakh et al., 2024). However, the application of AI often necessitates large quantities of labeled data, the preparation of which demands substantial time and effort (Paton et al., 2024). Creating accurate labels in agriculture often requires specialized knowledge, such as determining whether a pixel belongs to a specific weed type, further increasing the cost of the annotation process. Among annotation tasks, creating segmentation masks is particularly labor-intensive compared to deep learning tasks like classification or object detection.

As a subfield of image segmentation, every object instance of each class is assigned to one mask in instance segmentation. Such instances could be, e.g., single blood cells in a histological exam (Pal et al., 2024) or single maize kernels in maize ear phenotyping (Oury et al., 2022). Further applications of instance segmentation in plant phenotyping are the segmentation of the grapevine inflorescence (Moreira et al., 2025), or the counting of wheat ears (Dandrifosse et al., 2022). All these studies have in common that the training and testing of the proposed deep learning models rely heavily on high-quality ground-truth data.

Traditionally, annotation of segmentation masks involved pixel-wise labeling or drawing polygons to create precise masks (Castrejón et al., 2017). More recently, the adoption of AI-driven pre-labeling tools has emerged as a promising approach to accelerate the annotation process. Pre-labeling shifts the role of human annotators from manual labeling to refining AI-generated labels, reducing the effort required for data annotation (Shao et al., 2024). A suitable source for pre-labels in segmentation is the recently released foundation models segment anything model 1 (SAM 1) (Kirillov et al., 2023) and its successor, the segment anything model 2 (SAM 2) (Ravi et al., 2024). Both models were trained and successfully tested on various domains (Kirillov et al., 2023; Ravi et al., 2024). While SAM 1 only predicts masks on individual images (Kirillov et al., 2023), SAM 2 was designed to predict and track masks along video frames (Ravi et al., 2024). Both models feature an automatic mask generator (AMG), proposing masks without required input, and the prediction of masks based on input prompts such as bounding boxes or points (Kirillov et al., 2023; Ravi et al., 2024). Instead of using SAM 1 for pre-labeling, its prompting capabilities were often applied directly on different phenotyping tasks, such as the segmentation of potato leaves (Williams et al., 2024) or for phenotypical measurements on pumpkin, radish, and cucumber (Zhang et al., 2024).

In agriculture, images are typically collected from mobile platforms such as unmanned aerial vehicles (UAV) (Oehme et al., 2022; Rejeb et al., 2022), tractors (Boysen et al., 2023) or stationary plant phenotyping systems (Daviet et al., 2022; Kirchgessner et al., 2024). Here, one or more cameras move relative to one or more objects of interest, resulting in image sequences having varying overlap between images. In scenarios where such overlapping images need to be annotated, a human may need to annotate the same object on multiple images. Photogrammetry allows the orientation and merging of overlapping images, which is often applied in UAV imagery, resulting in orthomosaics (Rejeb et al., 2022). Annotators could, e.g., annotate masks on one combined orthomosaic instead of multiple original images. Yet orthomosaics can contain artifacts or distortions (Manzini et al., 2024), leading to bad annotations that might affect machine vision applications. In contrast, SAM 2’s mask propagation capabilities allow transferring masks from one consecutive image to the next without relying on photogrammetry. SAM 2’s design for video segmentation indicates robustness even on complex scenes, whereas photogrammetry assumes scenes do not move between captured images.

Although open-source annotation software, such as LabelMe, has already integrated SAM 1 as a pre-labeling tool (Wada, 2025), the effect of such tools on annotation time efficiency has not been studied on agricultural datasets. Similarly, to this date, no systematic optimization of AMG parameter selection has been conducted.

This study investigates the feasibility of using SAM 1 and SAM 2 as a pre-labeling tool to reduce instance segmentation annotation efforts on agricultural datasets. The study serves as a pathway to designing efficient annotation strategies, ranging from encoder selection to AMG hyperparameter optimization to the selection of suitable annotation tools. Therefore, the agricultural rapid annotation module based on segment anything models (ARAMSAM) is proposed, an open-source application built on top of SAM 1 and SAM 2. In this study, three key objectives are addressed:

- Evaluating the zero-shot performance of SAM 1 and SAM 2 encoders on previously unseen agricultural datasets;
- Optimizing AMG hyperparameters via a systematic grid search and analyzing its impact on annotation efforts;
- Quantifying the reduction in user interaction time of SAM-based methods as orchestrated by ARAMSAM and comparing them to polygon drawing as the previous standard method.

## 2 Materials and methods

### 2.1 Datasets

**Figure 1:**
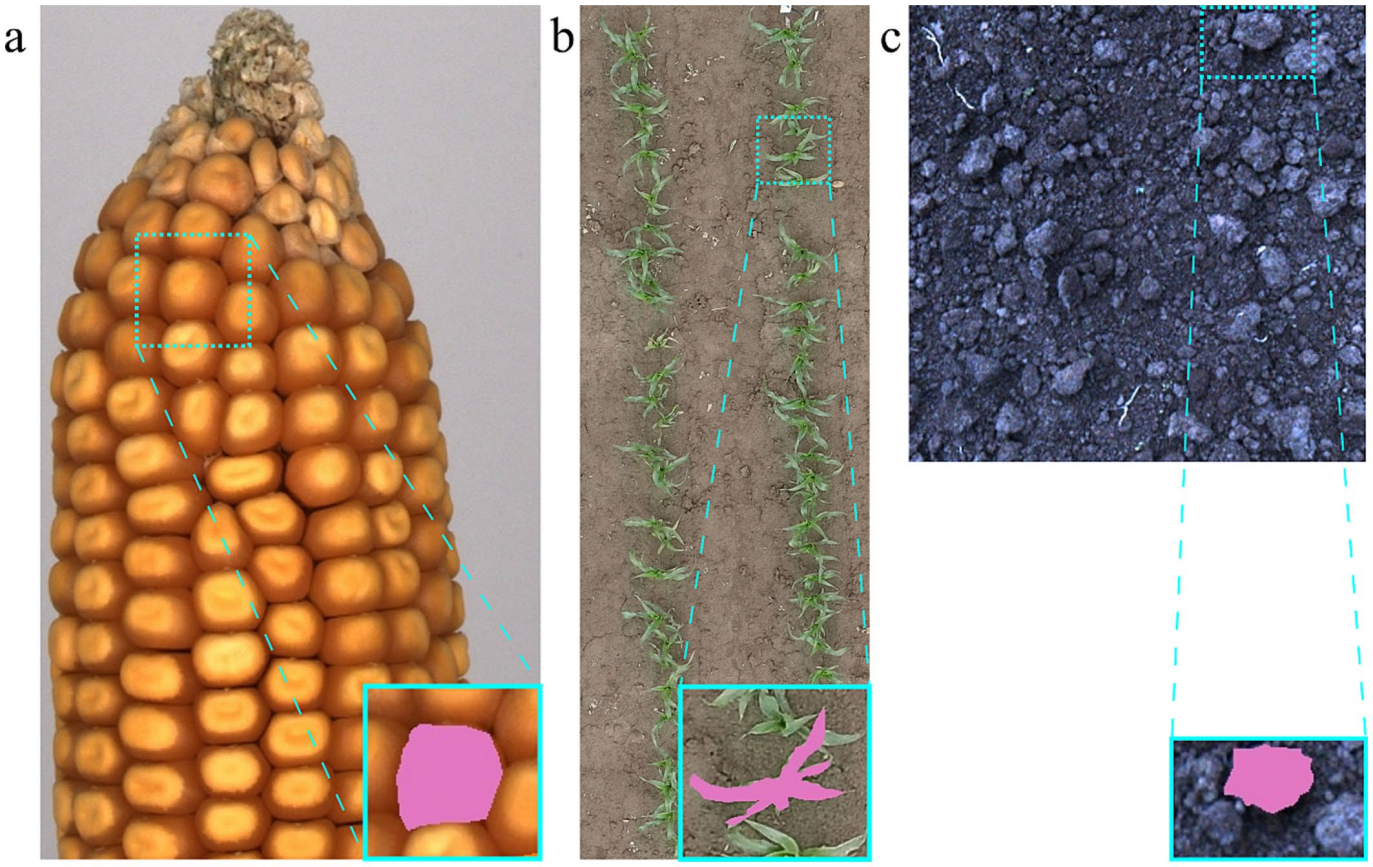
Image datasets: **(a)** Maize ear dataset (MED), **(b)** maize UAV dataset (MUD), **(c)** soil dataset (SOD); blue boxes highlight one segmentation instance as example, with the mask shown in pink.

Three datasets of RGB images, representing a range of common agricultural applications, were included in the experiments: (a) a maize ear dataset (MED), (b) a maize field UAV dataset (MUD) and (c) a soil surface dataset (SOD) (Figure 1).

Images of the MED, as shown in Figure 1a, were captured under controlled lighting conditions using an Alvium 1800 C-2050 camera (Allied Vision Technologies GmbH, Stadtroda, Germany) with a resolution of up to 5,376 × 3,672 pixels. The sensor was attached to a Kowa LM8FC24M lens (Kowa Company, Ltd., Nagoya, Japan) with an 8.5 mm focal length. Each ear was captured at 50 evenly distributed horizontal positions around the ear, rotating it stepwise by an angle *ω* of 7.1° between each image using a motorized rotating platform. To exclude the background area and to limit the annotation time per maize ear, the images were cropped to include only the upper half of the ear. Individual maize kernels represent the target object instances during later experiments.

The MUD comprises images of juvenile maize plants cultivated in two-row plots during a field trial (Figure 1b). These images were acquired in June 2024 using an UAV DJI M350 (SZ DJI Technology Co., Ltd., Shenzhen, China) operating at an altitude of 20 m above ground at an experimental farm of the University of Hohenheim in Stuttgart, Germany. The UAV was equipped with a DJI’s Zenmuse P1 sensor (8,192 × 5,460 pixels) and a P1 50 mm lens resulting in a ground sample distance of 3.1 mm/pixel. When conducting field experiments, the phenotypic data are usually collected per plot. To simulate the common postprocessing of experimental field data, the images were cropped to show one plot per image. The target instances are individual maize plants, and occluded parts are also included in the segmentation masks. Neither the MED nor the MUD has been published previously.

The SOD, as shown in Figure 1c, was collected after sowing with a power harrow sowing combination on ploughed fields around Stuttgart, Germany, in October and November 2022. The camera was mounted on the back of the machine and captured the images from a bird’s eye view. The images were captured with the SceneScan Pro-system of Nerian vision technologies (Allied Vision Technologies GmbH, Stadtroda, Germany) and were cropped to a size of 512 × 512 pixels. The ground sampling distance of the images is 1 mm/pixel. The dataset has been previously used to model the soil-machine interaction during secondary tillage by utilizing a deep learning model in Boysen et al. (2023). Individual soil clods represent the target instances for segmentation. Neither of the three datasets has been included in the training datasets of SAM 1 or SAM 2.

### 2.2 Encoder experiment

The architecture of SAM 1 (Kirillov et al., 2023) and its successor SAM 2 (Ravi et al., 2024) heavily rely on their image encoders for feature extraction. The encoder constitutes the largest part of the models and has a large influence on the resulting inference speed and segmentation quality. While SAM 1 employs the original vision transformers (ViT) by Dosovitskiy et al. (2020) as encoders, SAM 2 is based on less computationally complex hierarchical vision transformers (Hiera) (Ravi et al., 2024). For encoder selection, the performance of the three released encoders of SAM 1 (Kirillov et al., 2023) (ViT-B, ViT-L, ViT-H) was evaluated. Additionally, the four encoders of SAM 2 (Ravi et al., 2024) (Hiera-T, Hiera-S, Hiera-B+, Hiera-L) were evaluated in both their initially released version (SAM 2.0) and their updated version (SAM 2.1). To assess segmentation quality, the models were applied to all three datasets (MED, MUD, SOD). Therefore, 10 images and 10 object instances per image were randomly selected and annotated with the polygon feature of ARAMSAM (see chapter 2.4). The geometric median of the respective ground-truth masks, as defined by Vardi and Zhang (2000), was used as a positive point prompt for the model. A positive point prompt indicates to the model where to find a mask at the specific point in the image. Multiple points may be prompted to SAM to generate a mask. In contrast, negative points can be prompted to confine masks or exclude regions from a mask (Kirillov et al., 2023). To quantify segmentation accuracy while accommodating class imbalance between relatively small object instances and the background, the generalized dice score (*GDS*) (Sudre et al., 2017) implemented in Monai (1.4) (Cardoso et al., 2022) was used as a metric to evaluate the models’ performance. For this specific two-class case, the *GDS* can be defined for *N* pixels as:


$$\mathrm{GDS}=\frac{2\sum_{l=1}^{2}w_{l}\sum_{i=1}^{N}p_{i,l}\;g_{i,l}}{\sum_{l=1}^{2}w_{l}\sum_{i=1}^{N}(p_{i,l}+g_{i,l})},$$ (1)

Where $g_{i,l}\in \{0,1\}$ are the ground-truth labels and $p_{i,l}\in \{0,1\}$ are the predicted labels for the class l at pixel position i. The weight per class $w_{l}$ is defined as:


$$w_{l}=\frac{1}{{\left(\sum_{i=1}^{N}y_{i,l}\right)}^{2}},$$ (2)

Where $\;y_{i,l}$ is the one-hot encoded ground-truth label at pixel position ifor class l.

### 2.3 Automatic mask generator (AMG) hyperparameter optimization

Both SAM 1 and SAM 2 feature an AMG, which proposes masks without requiring a specific prompt input. Instead, a point grid is prompted internally, and predicted masks are filtered based on different tunable hyperparameters (Kirillov et al., 2023; Ravi et al., 2024). Both the density of the point grid and the strictness of mask filtering can be set via the hyperparameters. Definitions of the hyperparameters can be found in the docstrings of the “AutomaticMaskGenerator” classes within the SAM 1 and SAM 2 Python packages.

To optimize the AMG hyperparameters per encoder, a grid search over the given sets of hyperparameter variations was conducted on 10 previously annotated maize ear images, which show, other than images of the MED, only the maize ear center (Supplementary Figure S1). As can be seen in Table 1. The hyperparameter search space covered three different values for six hyperparameters, which covers 729 possible configurations in total. Some combinations did not produce any masks and even led to crashes of the SAM 2 package in 8 instances, which is why only 721 combinations are reported in this study. The specific faulty hyperparameter configurations can be seen in the ARAMSAM repository under the “preprint_v0.1” tag.

**Table 1:** Hyperparameter search space for automatic mask generators (AMG) of SAM 1 (ViT-H) and SAM 2.1 (Hiera-S).

Hyperparameter	Values	
	SAM 1 (ViT-H)	SAM 2.1 (Hiera-S)
points_per_side	{*32*, 64, **128**}	{***32***, 64, 128}
points_per_batch	{128}	{128}
pred_iou_thresh	{0.72, **0.8**, *0.88*}	{**0.72**, *0.8*, 0.88}
stability_score_thresh	{0.92, *0.95*, **0.98**}	{**0.92**, *0.95*, 0.98}
stability_score_offset	{0.7, ***1.0***, 1.3}	{**0.7**, *1.0*, 1.3}
crop_n_layers	{***0***, 1, 2}	{*0*, **1**, 2}
crop_n_points_downscale_factor	{***1***, 2, 4}	{***1***, 2, 4}

Italic values mark default values. Bold values mark optimal values.

The chosen search space aims to increase the number of proposed masks compared to the default configuration. At the same time, it also explores hyperparameter values close to the default configuration. Hyperparameter settings that were considered less significant were not tested but were kept at default values and are not listed in Table 1.

The *F_β_*-score with *β* = 2, weighing recall *R* four times as high as precision *P*, was chosen as a metric. Thereby, the production of more masks has been encouraged. The goal to increase the number of masks proposed by the AMG was driven by the assumption that manually discarding masks is less time-consuming than creating new masks manually for a human annotator. For precision and recall calculations, predicted and ground-truth masks were matched based on the intersection over union (*IoU*).

The *IoU* is defined as:


$$\mathrm{IoU}=\frac{\text{Area of Intersection}}{\text{Area of Union}}=\frac{|A\cap B|}{|A\cup B|},$$ (3)

Where the area of intersection is the overlapping region of two masks, and the area of union is the area covered by both masks combined.

Since predicted masks are not directly used for a downstream task but instead are used as annotations, true positives (TP) are defined at the ground-truth level. A ground-truth mask is counted as a TP if there exists at least one predicted mask with an Intersection-over-Union (IoU) greater than 0.8. This threshold was selected empirically based on preliminary tests that demonstrated sufficient mask quality. If a ground-truth mask is not matched with any predicted mask, it is counted as FN. False positives (FP) are predicted masks that cannot be associated with any ground-truth mask above the IoU threshold. Consequently, these definitions do not follow conventional one-to-one matching between predictions and ground truths. This is intentional since the annotation pipeline, in theory, allows a single predicted mask to be reused for multiple ground-truth instances, though this is very rare. Such a scenario would be a ground-truth instance that is occluded by another ground-truth instance. Here, the same predicted mask could be suitable to represent both the occluded instance and the instance on top. Thus, a single prediction could represent multiple TP.

Precision *P* is defined as:


$$P=\frac{\mathrm{TP}}{\mathrm{TP}+\mathrm{FP}},$$ (4)

while recall *R* is defined as:


$$R=\frac{\mathrm{TP}}{\mathrm{TP}+\mathrm{FN}}.$$ (5)

Thus, the *F_β = 2_*-score is defined as:


$$F_{2}=(1+2^{2})\;\frac{P\times R}{2^{2}\;P+R}=5\;\frac{P\times R}{4\;P+R}.$$ (6)

### 2.4 ARAMSAM software

ARAMSAM is a previously unpublished open-source image annotation software, developed in this study for instance segmentation and mask transfer from one overlapping image to the next. The software uses Python (3.10) (Van Rossum and Drake, 2009) and is based on publicly available packages of the Python universe. The software’s front end runs on PyQt6 (6.7) (Riverbank Computing, 2025) while the back end uses OpenCV (4.10) (Bradski, 2000) for conventional computer vision tasks, Pandas for data wrangling (2.2) (McKinney, 2010), PyTorch (2.4) (Paszke et al., 2019) for AI utilities and SAM 1 (Kirillov et al., 2023) and SAM 2 (Ravi et al., 2024) for semiautomatic zero-shot segmentation tasks.

**Figure 2:**
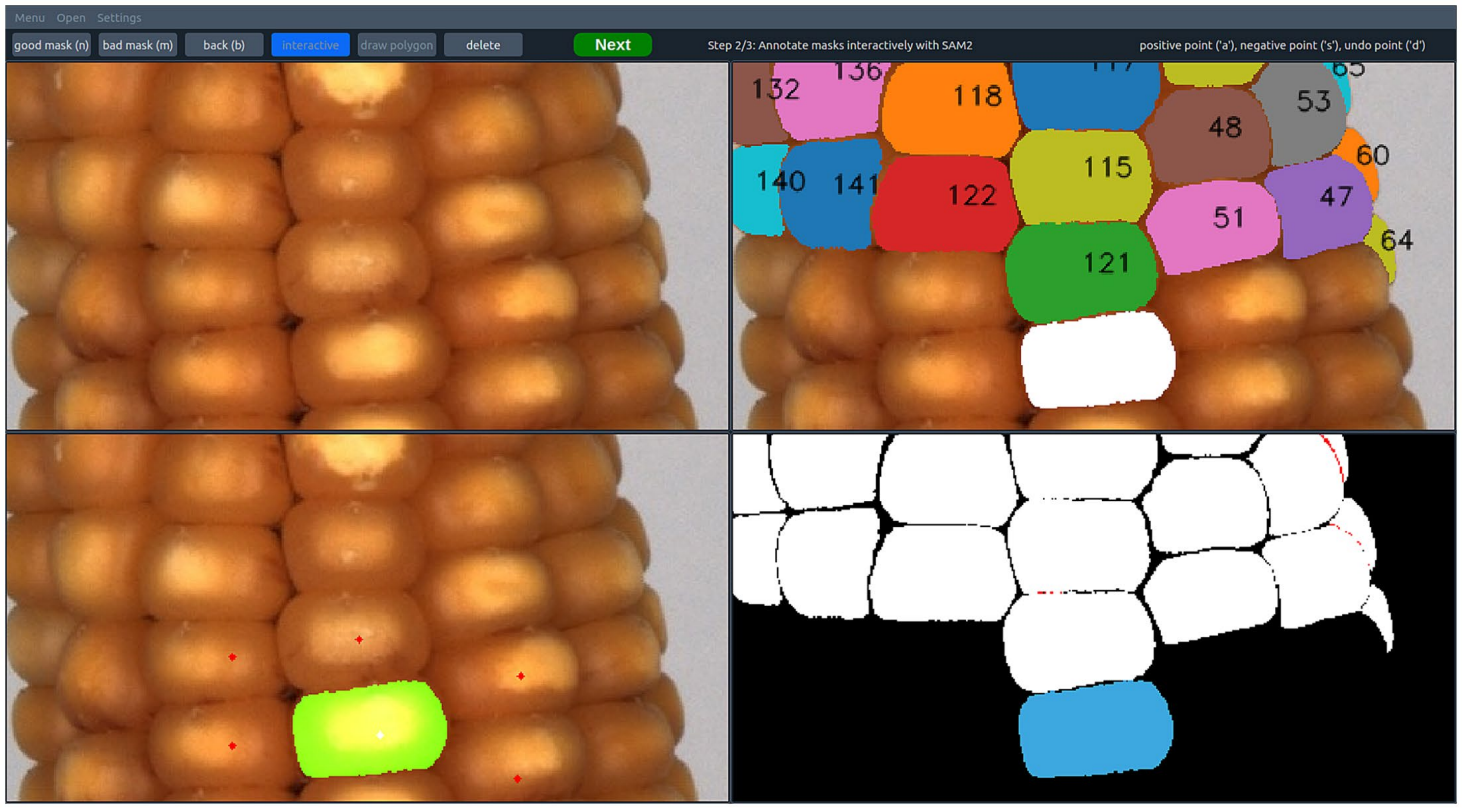
Overview of the ARAMSAM user interface. Top-left: Original RGB image. Top-right: Annotated masks overlaid on the original RGB image, with numbers indicating the mask ID. The white mask represents the preview generated by SAM 1/SAM 2. Bottom-left: Point view showing annotation prompts. Red points indicate negative prompts guiding SAM 1/SAM 2 to avoid these locations. White points indicate positive prompts guiding SAM 1/SAM 2 to include these locations. The preview mask is shown in green. Bottom-right: Annotated masks on black background. Overlapping mask areas are highlighted in red and the preview mask is shown in blue.

The user interface of ARAMSAM features a top bar with general settings and buttons for annotation actions (Figure 2). Below the bar, four freely selectable views of the image that is being annotated are visible. To ensure a comparable annotation process during the experiments, users were not allowed to change any settings themselves, and the views were predefined. The top-left view showed the original RGB image, and the top-right view showed the collection of previously annotated masks on the original. The bottom-right view showed the previously annotated masks in white, with overlapping mask parts being highlighted in red to avoid unintended overlap. The bottom-left view indicated points for both drawing polygons and interactively prompting with SAM 1 or SAM 2.

ARAMSAM enables creating segmentation masks with a set of tools to meet different demands for specific applications. The most common approach is to draw polygons around the object of interest. Another tool is the interactive prompting based on SAM 1 or SAM 2. Here, the user hovers the mouse over the image for real-time mask proposals (Figure 2). The image is embedded by the encoder beforehand. Multiple positive and negative points can be added to refine the proposed mask. Additionally, ARAMSAM employs AMG as a supplementary tool within SAM 1 and SAM 2. Thereby, masks are proposed sequentially, and the user’s task is to choose whether each mask represents an object of interest or should be discarded instead.

Furthermore, ARAMSAM includes functionalities to transfer masks from one image to another if the dataset contains consecutive, overlapping images. Since SAM 1 does not inherently feature mask propagation, a panorama-based algorithm is used to transfer masks. Here, image key points are detected by the ORB (oriented FAST and rotated BRIEF) feature detector (Rublee et al., 2011), which is based on FAST (features from accelerated segment test) (Rosten and Drummond, 2006) and BRIEF (binary robust independent elementary features) (Calonder et al., 2010). The key points are matched as shown by Brown and Lowe (2007). The resulting image orientation can be exploited to project bounding boxes of objects annotated on the first image to the following image. The projected bounding boxes are then prompted to SAM 1 with the second image. When using SAM 2 in ARAMSAM, masks are propagated by means of the mask propagation functionalities, which were originally designed for video object segmentation (Ravi et al., 2024). To load image sequences instead of video frames into the memory bank of SAM 2, a custom function was added to the original Python package.

### 2.5 User experiment

Fourteen experts in the field of agriculture were asked to annotate images of three randomly selected maize ears from MED in the ARAMSAM user interface. To familiarize participants with the software, each individual completed a tutorial demonstrating how to identify valid kernel masks and how to use all relevant tools for the experiment. During the experiment, every participant applied three different annotation methods to the same three initially selected maize ears. To mitigate potential learning effects over time, the order of these nine method–ear combinations was randomized for each user. An overview of the annotation methods is provided in Table 2.

**Table 2:** Overview of annotation methods in the user experiment.

Annotation method	Tools	Number of ears	Instance limit	Images per ear	Mask transfer
Polygon	Polygon annotation of highlighted maize kernels	3	3	1	–
SAM 1	1. Select AMG masks (image 1)2. Interactive prompting (image 1)3. Polygon drawing (image 1)4. Mask transfer and manual control (from image 1 to 2)5. Select AMG masks (image 2)6. Interactive prompting (image 2)7. Polygon drawing (image 2)		–	2	Panorama matching
SAM 2					Mask propagation

The users had to apply the three different annotation methods in separate steps of the experiment. SAM 1 and SAM 2 represent annotation methods consisting of multiple sequentially applied tools based on SAM 1 (Vit-H) and SAM 2.1 (Hiera-S), respectively. AMG: Automatic mask generator.

When participants were asked to use the polygon method, they were only required to annotate three maize kernels to avoid excessive effort. Before the experiment, three kernels per image were randomly selected and highlighted by bounding boxes, ensuring that all users annotated the same kernels.

For the annotation methods based on SAM 1 and SAM 2, users were given a fixed structure, as listed in the tool’s column of Table 2. These annotation methods with a fixed structure had to be applied to three image pairs that contain two consecutive images. The three image pairs were the same for SAM 1 and SAM 2, and the first image of each of the image pairs is used during the polygon method. The fixed structure is ordered from tasks requiring less interaction (e.g., selecting AMG masks) to tasks requiring more interaction (e.g., polygon drawing). If, e.g., all valid maize kernels of one image had been assigned a good mask created by the AMG, there was no need to apply interactive prompting or polygon drawing. After transferring masks from the first image to the second (either by the panorama approach or the SAM 2 propagation), the users were asked to check whether all masks had been transferred correctly and to remove invalid masks by clicking on them. The criteria for a mask being transferred correctly are assessed only by the quality of the mask on the second image. Individual maize kernels are not tracked back to the preceding image.

To evaluate whether annotation methods based on SAM 1 and SAM 2 can accelerate the annotation process of instance segmentation masks over previous standard methods, the drawing of polygons was used as a baseline. Since the annotation of the second image of an ear is influenced by the mask transfer capabilities of both the SAM 1 and the SAM 2 method, only the first image of an ear was taken for comparison across the polygon and both SAM methods. When the users were applying annotation methods based on SAM 1 and SAM 2, the users had to independently decide which object represented a valid maize kernel. To study the consistency of annotation decisions across different users, the annotation frequency per image pixel *f_a,px_* was defined as:


$$f_{a,\mathrm{px}}=\frac{N_{\text{assigned}}}{N_{\text{rounds}}},$$ (7)

Where *N*_assigned_ is the number of times a pixel has been assigned to a mask; *N*_rounds_ is the number of annotation rounds per image. With 14 users, each employing two annotation methods based on SAM 1 and SAM 2, the total number of annotation rounds per image was *N*_rounds_ = 28.

All experiments have been conducted on systems using a single RTX 3090 (NVIDIA Corporation, Santa Clara, USA) as a GPU.

### 2.6 Statistical analysis and data visualization

All statistical analyses were performed using R (4.3.2) (Team, 2025). Data wrangling and manipulation were carried out with Dplyr (1.1), Tidyr (1.3), and Tibble (3.2) from the Tidyverse universe (Wickham et al., 2019). Statistical tests and *post hoc* analyses were conducted using Rstatix (0.7) (Kassambara, 2019). Normalized metric result data [*p* ∈ (0,1)] was pre-processed with a logit transformation before applying statistical tests. Thereby, boundary constraints close to 0 or 1 and variance heterogeneity were tackled as shown in Zou et al. (2004). Repeated measures ANOVA results have been corrected by the Greenhouse–Geisser approach to mitigate sphericity of within-subject factors. Two-sided pairwise *t*-tests with Bonferroni correction have been conducted as post-hoc tests. Both the repeated measures ANOVA and the post-hoc tests rejected the H_0_-Hypothesis with a significance level of *α* = 0.05.

Python (3.10) (Van Rossum and Drake, 2009) and the Pandas package (2.2) (McKinney, 2010) were used for data preparation, followed by data visualization based on Matplotlib (3.10) (Hunter, 2007) and Seaborn (0.13) (Waskom et al., 2021). In all boxplots displayed in this study, the central box spans from the first quartile to the third quartile with a line inside marking the median. The whiskers extend to the smallest and largest data points within 1.5 times the interquartile range from the quartiles. Data points falling outside these limits are plotted individually as outliers.

### 2.7 Declaration of generative AI and AI-assisted technologies in the writing process

During the preparation of this study, the authors used ChatGPT 4-o (OpenAI, Inc., San Francisco, USA) to improve the writing. After using this tool/service, the authors reviewed and edited the content as needed and take full responsibility for the published article.

## 3 Results

### 3.1 Zero-shot performance of different SAM 1 and SAM 2 encoders

**Figure 3:**
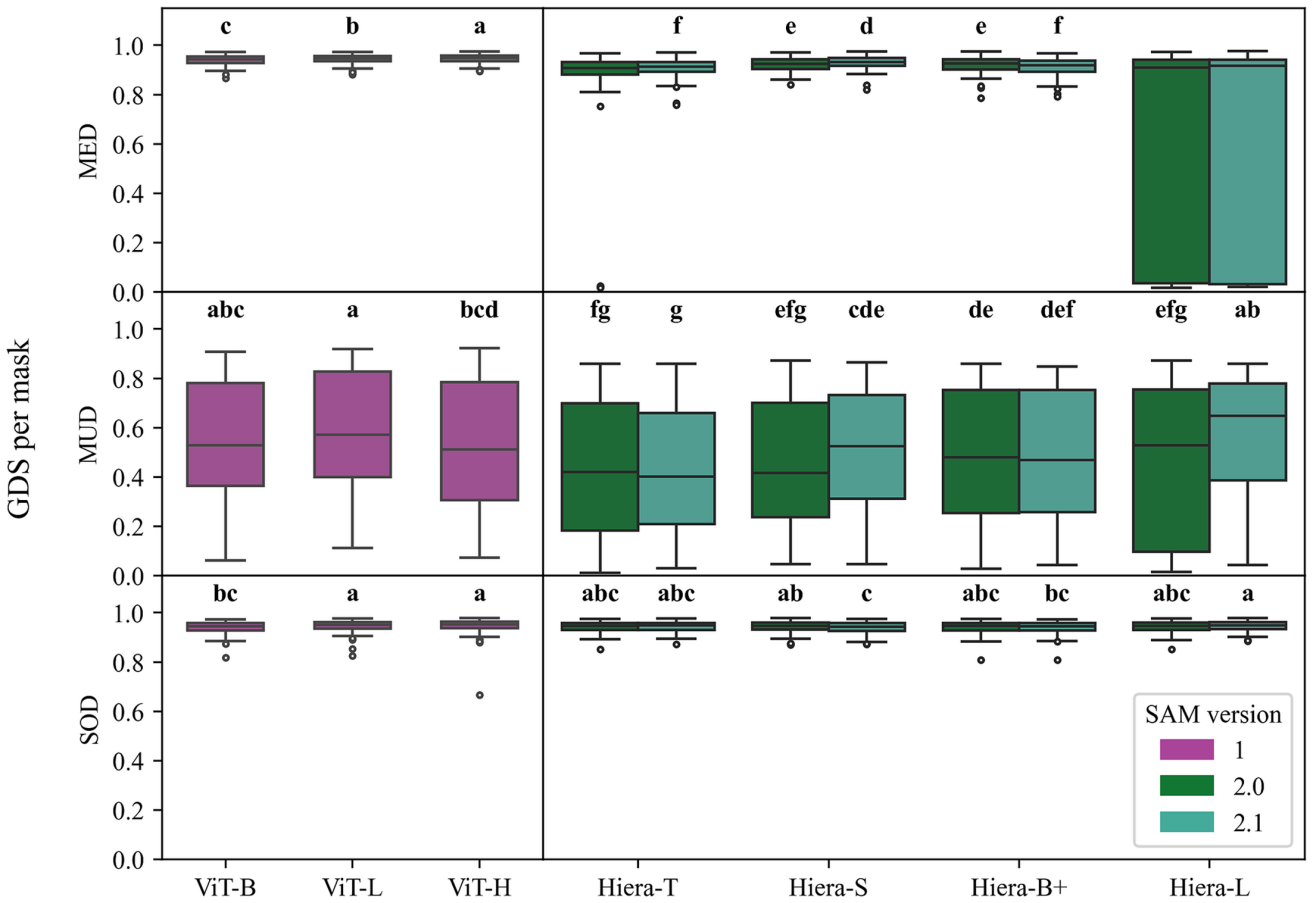
Segmentation quality of SAM 1 and SAM 2 across different encoder versions tested on the maize ear dataset (MED), maize UAV dataset (MUD), and soil dataset (SOD). The *x*-axis indicates the encoder versions, and the *y*-axis shows the generalized dice score (*GDS*) per mask. Significant differences are denoted by letters arranged from the highest mean *GDS* to the lowest (*α* < 0.05).

To evaluate mask quality as predicted by SAM 1 and SAM 2, all encoders have been applied on the agricultural datasets (MED, MUD, SOD), with a single point (the geometric median) for each of the 100 ground-truth masks—a comparison between the predicted mask and the ground-truth mask results in the *GDS*. In Figure 3, the resulting *GDS* of the encoder experiments are displayed for the MED, MUD and SOD dataset (from top to bottom). The scores range from 0 to 1 and are displayed as a boxplot indicating the distribution of the quartiles. All encoders achieve relatively high mean *GDS* for both the MED (0.87) and the SOD (0.94) compared to the MUD (0.50). These results were expected as the ground-truth masks of both the MED and SOD resemble compact round objects, whereas the plant objects in the MUD are complex and partially overlap with neighboring plants.

To compare the results of all individual encoders, a repeated measures ANOVA was conducted per dataset. Significant effects across encoders were revealed for all datasets, with the test results being *F*(2.96, 292.92) = 165.48, *p* < 0.001, for the MED, *F*(6.23, 616.59) = 17.60, *p* < 0.001, for the MUD, and *F*(4.44, 439.61) = 7.71, *p* < 0.001, for the SOD. A pairwise *t*-test was used as a post-hoc test (*α* < 0.05) with significant differences shown in Figure 3. Two encoders not sharing a letter achieved significantly different performance on the respective dataset. The alphabetical order of the letters indicates the performance ranking from “a” the best to “g” the worst performing group.

The Hiera-T encoder of SAM 2.0, as well as both versions (SAM 2.0 and SAM2.1) of the Hiera-L encoder, had to be excluded from statistical tests of the MED since normality of the data could not be assumed. This is indicated by the absence of a letter. Since these three encoders apparently do not show good performance, they are irrelevant for further experiments and thus can be excluded from statistical tests. This performance test of the encoders was conducted to identify the best encoders for later experiments.

Notably, at least one encoder of SAM 1 belongs to the significant letter “a” for all three datasets. Moreover, all encoders of SAM 1 show significantly higher *GDS* per mask than any encoder of SAM 2 when tested on MED, indicating stronger or equal performance of SAM 1 on all datasets when compared to SAM 2.

The compared encoders vary regarding their network architecture and the number of parameters (Kirillov et al., 2023; Ravi et al., 2024). Thus, the largest ViT-H (SAM 1) and Hiera-L (SAM 2) encoders are less computation-efficient than their smaller counterparts. Figure 3 indicates that the smallest encoder types of both SAM 1 (ViT-B) and SAM 2 (Hiera-T) show significantly lower *GDS* than the larger encoders. However, the largest encoder types (ViT-H, Hiera-L) do not perform significantly better than the medium-sized encoders. Interestingly, the updated encoders of SAM 2.1 only show a significantly higher *GDS* than their predecessors for the Hiera-S encoder at MED and for the Hiera-L encoder at the SOD. In contrast, the old SAM 2.0 encoders achieved a significantly higher *GDS* for the Hiera-B+ encoder at MED and the Hiera-S encoder at the SOD. These results indicate no improvement of the updated SAM 2.1 encoders over the initially released ones on the proposed agricultural use cases.

### 3.2 Automatic mask generator (AMG) hyperparameter optimization

The following experiments focused on images of the MED because maize ears are complex, round objects that were captured from different angles. Thus, object tracking was expected to be a more challenging task than with images of the MUD and SOD where all image planes are parallel to another and to the soil surface. Moreover, only the best performing encoders of SAM 1 (ViT-H) and SAM 2.1 (Hiera-S) according to *GDS*, as achieved on the MED, were selected for optimizing hyperparameters of the AMG. The best performing hyperparameters according to the mean *F_2_* over all images have been identified on a new subset of the MED, where ground-truth masks for all kernels have been annotated manually (Table 1).

Within the optimal settings of SAM 1 (ViT-H), the identified values for the hyperparameters *points_per_side* and *pred_iou_thresh* are increasing the number of proposed masks compared to the default values but in contrast, the setting of *stability_score_thresh* is applying a stronger filter to the proposed masks than the default value. However, the settings of hyperparameters for SAM 2.1 (Hiera-S) increases the number of masks by a reduced value of *pred_iou_thresh*, by an increased number of *crop_n_layers* and with reduced *stability_score_thresh* as well as reduced *stability_score_offset*. Notably, the optimal hyperparameters of SAM 2.1 (Hiera-S) include no deviation from the default settings that would reduce the number of masks. These different optimal settings highlight the models’ network architectural differences as revealed on the MED.

**Figure 4:**
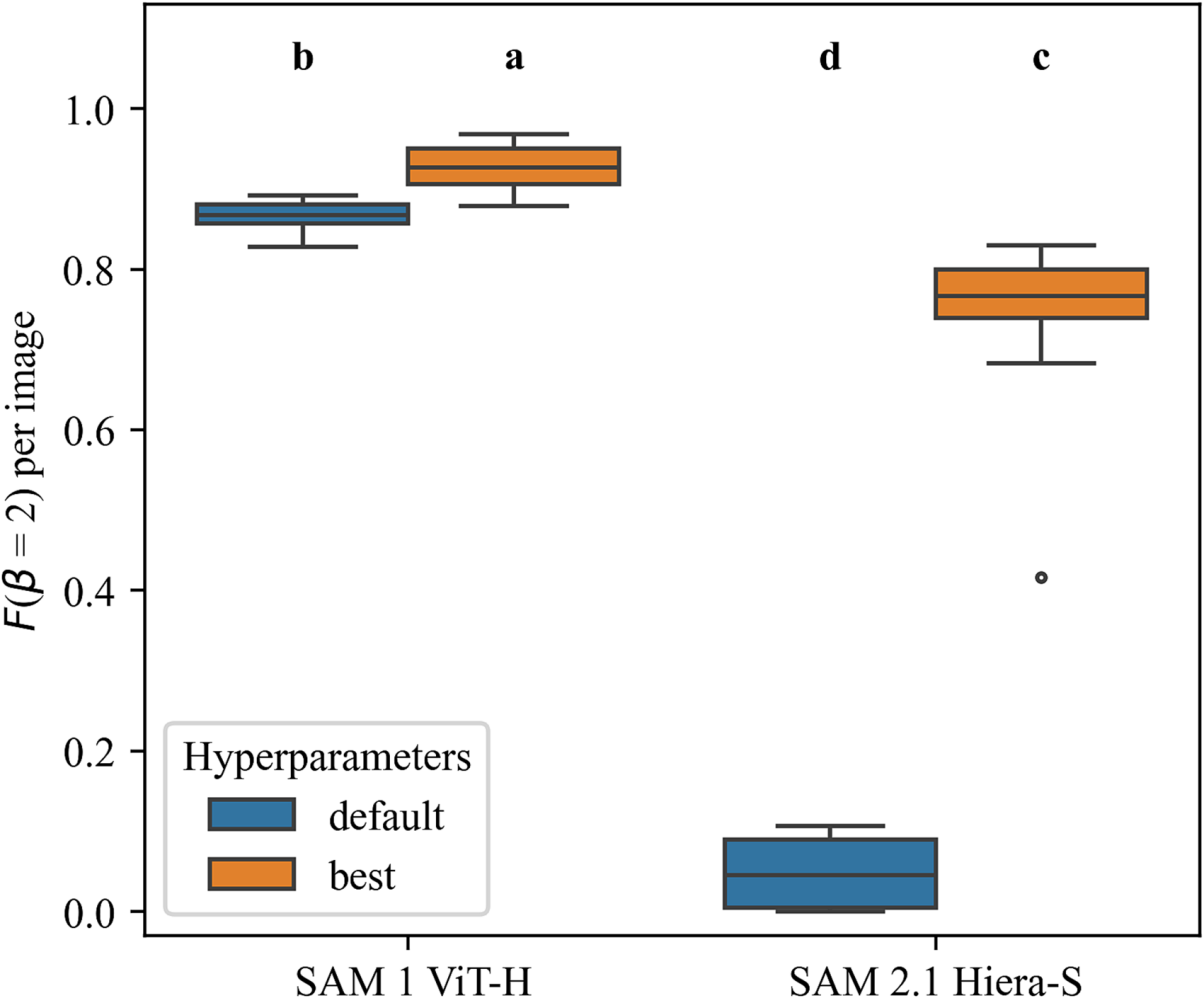
Mask quality predicted by automatic mask generators (AMG) before (default) and after hyperparameter optimization (best). The models were evaluated per image using F2 calculated from the number of predicted masks that matched ground-truth masks. Significant differences (pairwise *t*-test, *α* < 0.05) are indicated by different letters.

Figure 4 shows the *F_2_*-score of the selected SAM 1 encoder (ViT-H) and SAM 2.1 encoder (Hiera-S) per test image. A significant effect of the encoders and hyperparameters was revealed by repeated measures ANOVA (*F*(1.04, 9.4) = 31.27, *p* < 0.001). The AMG of both SAM benefits significantly from the hyperparameter optimization compared to the default configuration. While SAM 1 (ViT-H) improved from a mean *F_2_*-score of 0.87–0.93, SAM 2.1 (Hiera-S) improved from 0.05 to 0.74.

Again, the encoder representing SAM 1 (ViT-H) performs significantly better than SAM 2.1 (Hiera-S). Moreover, the SAM 2.1 (Hiera-S) encoder achieved an especially low *F_2_* on one image, depicted as an outlier in Figure 4. Conversely, the selected SAM 1 (ViT-H) encoder does not show any outliers, indicating more stable performance on the MED.

### 3.3 User experiment

**Figure 5:**
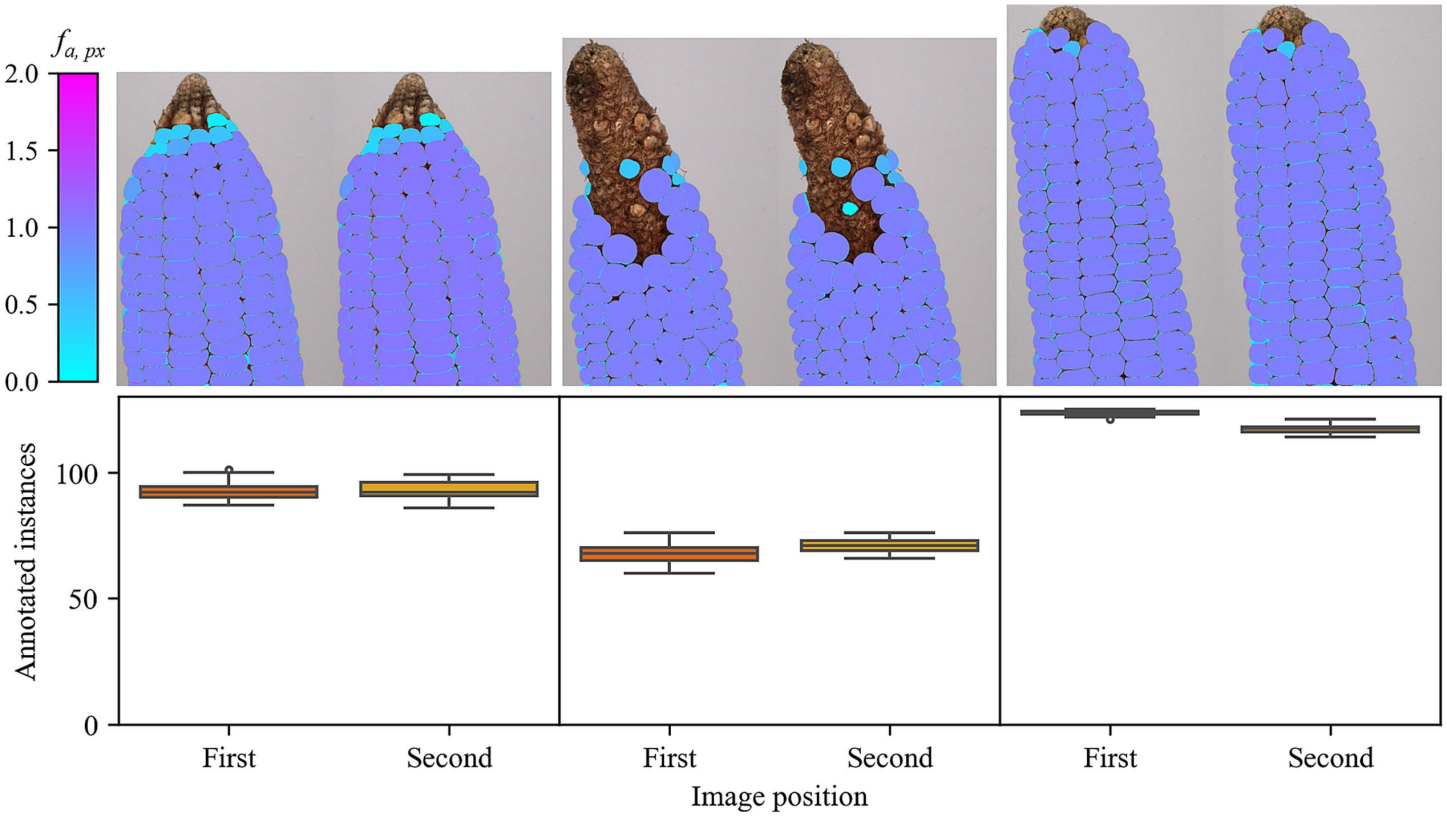
Annotation decisions of 14 users for three maize image pairs. Top: Relative frequency a pixel has been assigned to a mask (*f_a, px_*) for the total number of annotation rounds for the first (*ω* = 0°) and second rotated image (*ω* = 7.1°) of a maize ear. Only pixels assigned to a mask more than once are included. Bottom: Number of kernel instances annotated per image over all applied methods. The polygon method is excluded from the figure.

After identifying the best-performing encoders and the optimal AMG hyperparameters for SAM 1 (ViT-H) and SAM 2 (SAM 2.1 Hiera-S), respectively, a user experiment based on ARAMSAM was conducted with these configurations. All users had to apply annotation methods based on SAM 1, SAM 2, and the drawing of polygons. Thus, each image has been annotated by each user in multiple rounds. While, the objects of interest were highlighted during the polygon method, when using the other methods, the participants had to decide on their own which objects represent valid kernels according to the instructions they were given in the tutorial. The annotation decisions for all three image pairs are shown in Figure 5. As shown in the bottom row of Figure 5, the number of annotated instances (maize kernels) varied little across different users. The highest standard deviation of annotated instances per image (3.9 kernels) is observed for the first image of the left image pair. Consistent annotations across users are also confirmed by the top row of Figure 5, where most kernels have been annotated at annotation frequency *f_a, px_* close to 1.0. Nevertheless, lower *f_a, px_* can be observed for kernels in the area of the infertile tip, as shown in the left and the center image pairs. An enlarged view of the leftmost image is displayed in the supplementary data (Supplementary Figure S2). The rare occurrence of pink color on some kernel edges indicates that overlapping masks were assigned to neighboring kernels, which represents under-segmentation. Conversely, the light-blue color on kernel edges indicates that the assigned kernel masks were too small and did not cover the entire kernel. This over-segmentation can be observed on all ear images (Figure 5). Yet, both under-segmentation and over-segmentation usually cover a few pixels, which should have a minor influence on applications such as phenotyping.

**Figure 6:**
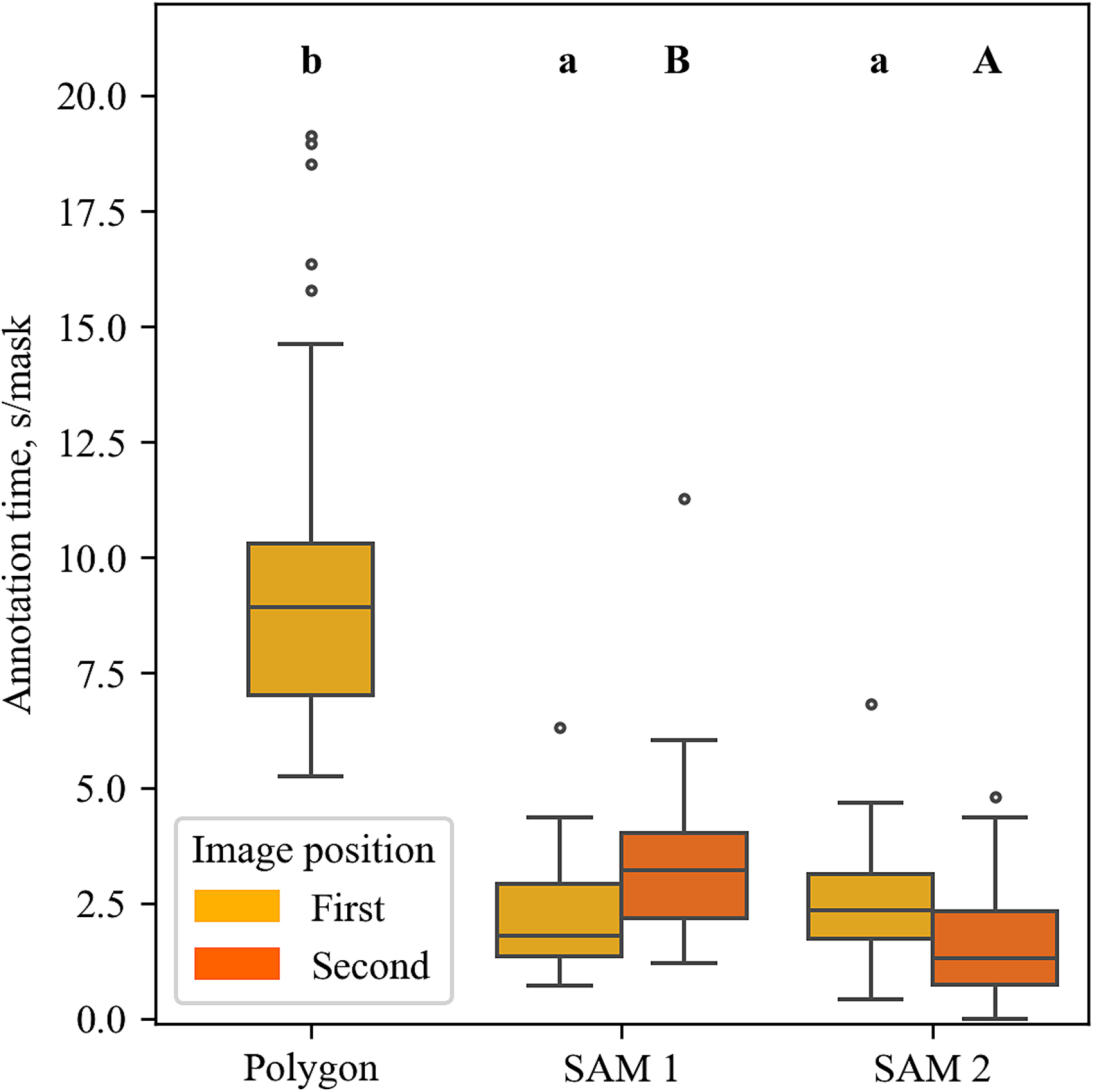
Annotation time per mask across different methods (X-axis). Significant differences are indicated by lowercase letters across first images and by capital letters across second images (pairwise *t*-test, *α* < 0.05).

In Figure 6, the annotation time per mask is displayed for the SAM 1 and SAM 2 approaches for both the first and the second of the consecutive images, as well as for the polygon method for the first of the consecutive images. Each boxplot contains 42 data points (14 users times 3 images). A significant effect of the annotation method on the annotation time per mask was revealed by a repeated measures ANOVA (*F*(1.11, 14.43) = 64.70, *p* < 0.001). The subsequent post-hoc test shows significant differences between the polygon method and both SAM methods (indicated by different letters). Accordingly, a significant difference in annotation time was observed with 9.7 s/mask for the polygon method. The approaches based on SAM 1 and SAM 2 took 2.1 and 2.6 s/mask, respectively. However, no significant differences between SAM 1 and SAM 2 were observed in the first images.

In the next step, masks from the first images were transferred to the second images either by the panorama-based algorithm (SAM 1) or mask propagation (SAM 2). On the second images a significant difference between SAM 1 and SAM 2 could be shown by both the ANOVA (*F*(1, 13) = 24.177, *p* < 0.001) and the post-hoc test. The latter indicated a significantly lower annotation time of SAM 2 when compared to SAM 1 (Figure 6). Strikingly, the mean of SAM 1 on the second image (3.3 s/mask) is higher than that on the first image (2.1 s/mask).

**Figure 7:**
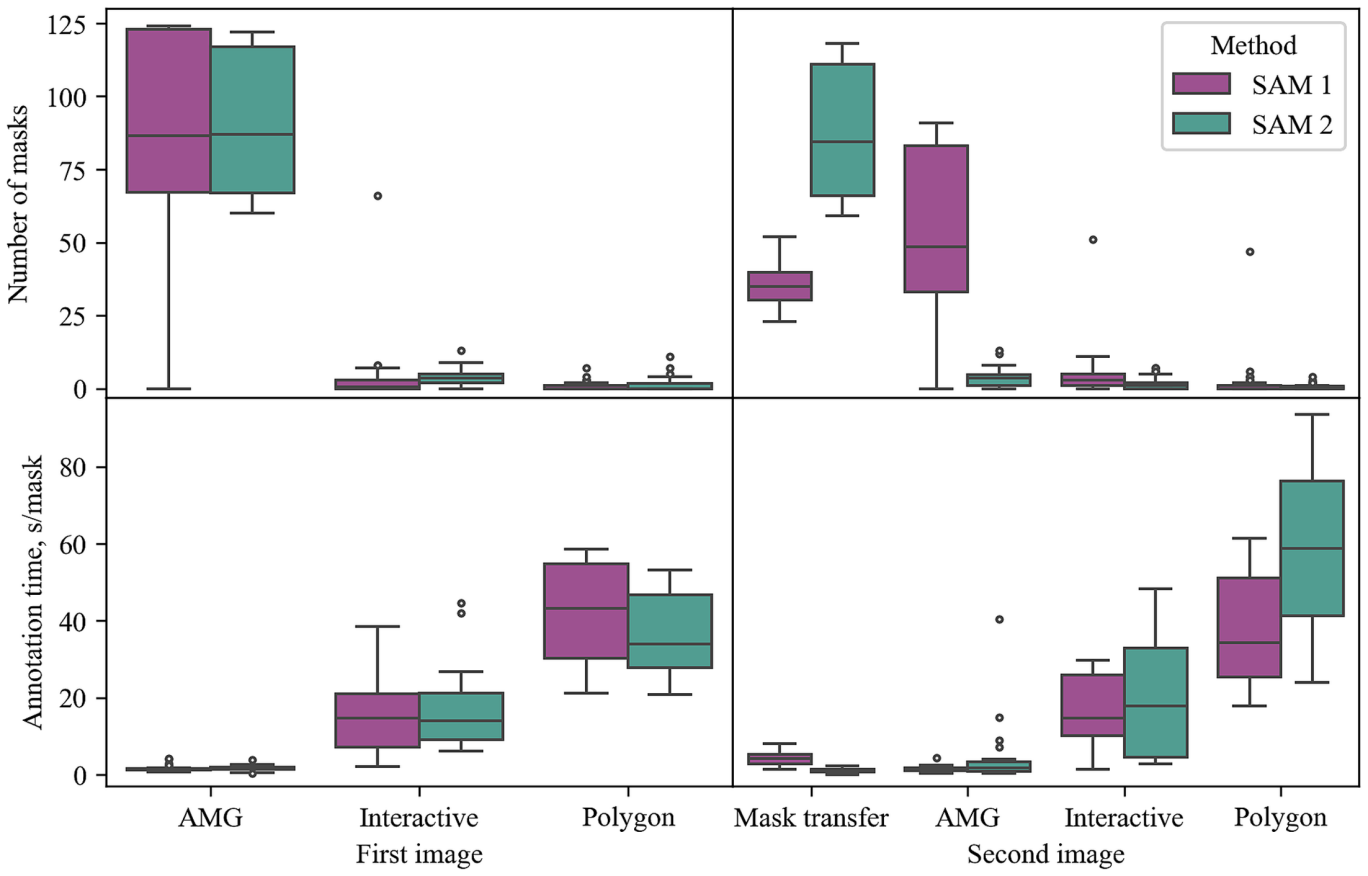
Applied tools within annotation methods. Data points represent one annotation round per user. Time (s/mask) is based on *the* mean temporal distance between individual masks when more than one mask was annotated per tool. AMG: Automatic Mask Generator.

Figure 7 depicts the number of masks generated and the annotation time (s/mask) for each tool. The AMG was the most applied tool of the SAM 1 method on the second image (56.0%) (Figure 7), suggesting that the majority of masks were not transferred correctly from the first to the second image. Likewise, the AMG of SAM 1 and the AMG of SAM 2 were also the predominant tools on the first images, where users could not benefit from transferred masks. Here, AMG was the origin of 95.8% (SAM 1) and 94.9% (SAM 2) of the selected masks (Figure 7). Thus, the users’ annotation behavior when transferring a mask from panorama matching (SAM 1) was similar to starting from a new image. This shows that SAM 1 did not benefit from the previous annotations and the panorama-inspired method for mask transfer of SAM 1 appears to not be suitable for the MED.

However, the SAM 2 method on the second image represents the fastest method over both image positions by requiring 1.6 s/mask on average. This is also supported by mask transfer being the predominant origin of masks created by the SAM 2 method on the second image (94.0%) (Figure 7). To determine whether the SAM 2 method, benefiting from masks propagated from the previous image, allows significantly faster annotation time than applying SAM 1 directly on the first image, a one-sided pairwise *t*-test was conducted. Here, the time per mask has been averaged over the images. The test results show significantly faster annotation times of SAM 2 (*t*(13) = 2.03, *p* = 0.032), suggesting that applying SAM 2 with mask propagation on image sequences of the MED is the fastest of the proposed methods.

## 4 Discussion

### 4.1 Comparing zero-shot performance of SAM1 and SAM2

Throughout this study, SAM 1 and SAM 2 were compared according to mask quality per prompt on three datasets (MED, MUD, SOD), mask coverage of the respective AMG on one dataset (MED), and temporal annotation effort for users on one dataset (MED). SAM 2 did not outperform its predecessor in any of these disciplines when applied to single images. Architecture-specific problems became apparent during the encoder experiment, where both Hiera-L encoders and the initial Hiera-T (2.0) encoder of SAM 2 are predicting the entire ear as a single mask instead of individual kernels in multiple instances (Figure 3). These problems specific to a certain encoder size highlight the need for proper model selection depending on the data.

While this study covered only agricultural use cases with RGB data, Sengupta et al. (2025), compared SAM 1 and SAM 2 on medical datasets covering both RGB and grayscale data. The authors showed that SAM 2 does not consistently perform better than SAM 1, which appears to be independent of image data type. However, SAM 2 achieved higher mask accuracy metrics in segmenting solar panels on remote sensing data (Rafaeli et al., 2024), in contrast to this study especially SAM 2.1 (Hiera-L) outperformed SAM 1 (ViT-L). Ravi et al. (2024) showed an improvement of SAM 2 for zero-shot performance (single images) on the most of 37 datasets from multiple domains. However, the 37 datasets barely focus on agriculture, besides the PPDLS (plant phenotyping datasets leaf segmentation) (Minervini et al., 2016), containing plant phenotyping data. Here, the performance of SAM 2 showed a delta of −4.8 mIoU (mean intersection over union) compared to the performance of SAM 1 (Ravi et al., 2024), indicating a setback in the performance of SAM 2 over its predecessor. The findings from Ravi et al. (2024) on the PPDLS, together with the results presented here, suggest that SAM 2 does not represent an improvement over its predecessor regarding zero-shot performance on single images of agricultural datasets.

Yet, Ravi et al. (2024) and Rafaeli et al. (2024) showed better performance of SAM 2 compared to SAM 1 in most domains. This is especially noteworthy because of SAM 2 enormous time savings in computation. Due to the smaller hierarchical image encoders, SAM 2 is six times faster than SAM 1 (Ravi et al., 2024). The architectural differences could be considered as a reason why the optimal parameters of the selected encoders of SAM 1 and SAM 2 differ greatly. Another benefit of SAM 2 is its ability to propagate masks from one image frame to the next. Incorporated in ARAMSAM, this feature accelerated the annotation time from 2.1 s/mask to 1.6 s/mask, by about 23% (Figure 6). Despite SAM 2 not showing improved mask quality compared to SAM 1, the mask propagation capability, as well as the more efficient model architecture, can lead to SAM 2 accelerating annotation times and saving human labor. Especially on image sequences, SAM 2 is considered the most suited method for annotation with ARAMSAM on the MED.

The positive results of our experiments are valid for the controlled environments represented by the datasets that all originated from the same geographical region. Transferring the results to less controlled conditions would expose the models to challenging properties like the tempo-spatial variations of environments, e.g., through weather effects or seasonal growth. These include background disturbances, partial or full occlusion of relevant object features or complete objects, different object sizes, rotations, or deformed objects, e.g., through wind and illumination changes due to varying daylight conditions (Song et al., 2025). Furthermore, the process of image acquisition (sensor type, motion blur, processing algorithms) also affects the image quality, which is important for successful use with deep learning methods (Dodge and Karam, 2016). Despite the limited variability of datasets in the experiments of this study, a certain robustness of ARAMSAM to new conditions would be expected, since SAM 1 and SAM 2 are foundation models that have been trained on large and diverse datasets (Ravi et al., 2024).

### 4.2 Parameter optimization of automatic mask generator (AMG)

To our knowledge, this is the first study doing a hyperparameter optimization on the automatic mask generator of both SAM 1 and SAM 2. In the user experiment the AMG was the most used tool for both image positions of the SAM 1 method and the most used tool of the SAM 2 method on first images (Figure 7). Although it should be noted that the here proposed structured experiment design fostered the usage of the AMG being used by the participants in first or second position, the capability of the AMG to cover more than 95.8% (SAM 1) and 94.9% (SAM 2) of the valid maize kernels is remarkable. Of all tools, the AMG of both SAM 1 and SAM 2 showed the lowest annotation time on the first images, taking only taking 1.5 s/mask and 1.7 s/mask, respectively (Figure 7). At the same time, the data shows a low standard deviation of 0.7 s/mask (SAM 1) and 0.6 s/mask (SAM 2), manifesting the tools’ reliability. As demonstrated in Figure 4, the AMG of both SAM 1 and SAM 2 benefited greatly from the hyperparameter optimization. Especially the results of SAM 2, improving the *F_2_* by more than 14 times, underline the importance of hyperparameter optimization when applying the AMG.

Conversely, the AMG could propose a large share of useless masks in scenarios where only a few objects of interest exist in one image. However, in crowded scenes where most objects represent object of interest like in the MED, the AMG can be especially useful. Therefore, exploiting the potential of this powerful tool by hyperparameter optimization is an important contribution to accelerating the annotation of segmentation datasets.

### 4.3 Time savings by ARAMSAM orchestrating SAM-based annotation tools

Applying annotation tools based on both SAM 1 and SAM 2 clearly outperformed the polygon method representing a former state of the art method for annotation of segmentation masks. For single images, the annotation time per mask is accelerated by 4.6 times for SAM 1 and 3.7 times for SAM 2. When applying SAM 2 with mask propagation on image sequences, the acceleration increases by a factor of 6.1 compared to the polygon method. Yet, it should be noted that the panorama-based mask transfer of masks proposed by SAM 1 did slow down the annotations by factor 1.6 compared to applying SAM 1 without any mask transfer. This highlights the difficulties of mask transfer even on highly overlapping images and indicates that this method was not suitable for mask transfer on the MED. Likely, the panorama-based mask transfer would have performed better on image sequences moving in a linear direction instead of the circular rotation presented by the MED. A more computation-intensive alternative could be a structure from motion (Schonberger and Frahm, 2016) based approach. Like the panorama algorithm, structure from motion matches multiple key points from overlapping images. In contrast to panorama stitching, the key points as well as the camera positions are oriented in 3D space, which would allow mask transfer even on irregularly shaped objects such as maize ears. However, structure from motion requires multiple images and sophisticated computation hardware to be applied in an edge scenario like image annotation (Schonberger and Frahm, 2016).

The SAM tools implemented in ARAMSAM can save a tremendous amount of labor on the MED dataset compared to polygon drawing. Since SAM 1 and SAM 2 were trained and successfully tested on various domains (Kirillov et al., 2023; Ravi et al., 2024), ARAMSAM has the potential to further accelerate the annotation process in other domains than the MED. Yet, it should be noted that the maize kernels represent rather simple objects with regular, round shapes and clear edges. While demonstrating how SAM 1 and SAM 2 orchestrated by ARAMSAM accelerate annotation speed, this study does not compare ARAMSAM to other public annotation software. Our findings suggest that other software incorporating SAM 1 and SAM 2 would benefit from similar gains in annotation speed.

Increasing the annotation efficiency can be especially relevant in fields where human expert knowledge is required. Besides plant phenotyping, one such field would be medicine, where, e.g., radiologists have to label malignant tumor tissue on CT scans (Zhu et al., 2024). Also, the example of maize kernels (MED) showed the challenges of qualified decision-making. Although all participants saw the same example masks of valid and invalid maize kernels during a tutorial, the decision on which of the top kernels shown on the left and center pair of maize ears in Figure 5 represent valid kernels was ambiguous. In a scenario where, e.g., the length of the infertile tip of a maize ear should be measured (Oury et al., 2022), inconsistent decisions on which kernel to label as valid or fertile could have a crucial impact on the results. However, the agricultural experts that participated in this study were not specifically experts for maize ear phenotyping. Even for professionals in that field, borderline cases and human errors cannot be ruled out for any annotator.

Despite the tutorial covering all functionalities of ARAMSAM, human errors could be observed at the user experiment depicted as outliers in Figures 6, 7. A few participants appeared to be stuck in certain steps of the experiment, leading them to spend exceptionally long in some annotation tools. Since these outliers indicate a certain complexity of the experiment and do not represent measurement errors, they were included in the statistical analysis. However, the rare occurrence of these outliers shows that only few users encountered these difficulties and most of them were able to learn ARAMSAM quickly.

The integration of the AMG, interactive prompting and mask transfer options for both SAM 1 and SAM 2 as well as polygon drawing as a baseline demonstrates the versatility of ARAMSAM in annotating segmentation datasets. Since the source code of the software will be published along with this paper and since ARAMSAM is completely written in Python, it will be relatively easy to adapt to specific demands. Besides the here conducted annotation experiments ARAMSAM, can be directly used for annotating single images and image sequences for the purpose of training a specific AI model. Such image sequences could include videos, overlapping neighboring images or slices of 3D-data such as CT-Scans or polygon meshes. Also using ARAMSAM directly for measurements of objects in images would be feasible, if intrinsic as well as extrinsic camera parameters and the distance to the object of interest are known.

The development of novel AI-based phenotyping solutions could benefit greatly from accelerated mask annotation based on ARAMSAM. Although Ravi et al. (2024) state that mask propagation would suffer from crowded scenes with many object instances, the findings of this study on the MED suggest successful mask propagation in most cases. On average, 94.0% of the masks annotated on the second images originated from mask propagation (Figure 7). It should be noted that the maize ears are rotated by only 7.1°, leaving a substantial overlap between images to be exploited by SAM 2. Yet, this overlap might be smaller than that of consecutive video frames, for which the SAM 2 mask propagation was designed for (Ravi et al., 2024). How far this overlap can be reduced remains an open research question. For both annotation tasks and zero-shot applications, a falsely propagated mask can have a negative impact. However, an overlap of around 80% is, e.g., common in UAV missions for creating digital surface models based on photogrammetry (Oehme et al., 2022). This substantial overlap suggests potential for mask propagation with SAM 2 on field image data captured by UAV.

## 5 Conclusion

In this study, the potential of both SAM 1 and SAM 2 to accelerate the annotation of segmentation masks as orchestrated by ARAMSAM was evaluated. The annotation time was accelerated by up to 4.6 times (to 2.1 s/mask) with SAM 1 on single images and up to 6.1 times (to 1.6 s/mask) with SAM 2 on image sequences when compared to polygon drawing, representing remarkable time savings. Moreover, the results on zero-shot performance and from user experiments applying SAM 2 on single images suggest, in accordance with the literature, that SAM 2 represents no improvement on agricultural datasets over SAM 1. In future research on annotation methods in the agricultural domain, finetuning SAM 2 could further accelerate the annotation process.

Furthermore, the importance of hyperparameter optimization of the AMG of both SAM 1 and SAM 2 was demonstrated. The *F_2_*-score of predicted masks by SAM 2 when matched to ground-truth masks has been improved by more than 14 times (from 0.05 to 0.74) via grid search for optimal hyperparameters. Moreover, efficient optimization techniques covering larger search spaces such as evolutionary algorithms could be applied in future studies using the AMG.

ARAMSAM, which was developed in this study, is a flexible framework that provides user-friendly access to tools based on SAM 1 and SAM 2. However, the annotation acceleration of SAM 1 and SAM 2 should be further quantified on more diverse and challenging agricultural datasets than those presented in this study. Furthermore, the annotation capabilities of ARAMSAM remain to be compared to other public annotation software in a future study. Nevertheless, built on a Python foundation, ARAMSAM is easily extendable with custom code, allowing researchers to tailor its functionalities to specific needs. Future implementations may include the ability to assign classes to segmentation masks, enriching the software’s annotation capabilities. Moreover, ARAMSAM could be integrated with active learning approaches by incorporating pretrained models, which would facilitate the iterative refinement of model performance.

Overall, ARAMSAM, as being published along with this study, is a powerful software solution that integrates the ground-breaking functionalities of both SAM 1 and SAM 2, while also possessing the potential to evolve and make a significant impact on machine vision in agriculture and beyond.

## Data Availability

The datasets presented in this study can be found in online repositories. The names of the repository/repositories and accession number(s) can be found at: https://github.com/DerOehmer/ARAMSAM/releases/tag/preprint_v0.1.

## Supplementary material

Supplementary Figure S1 Example of maize ear images used for hyperparameter optimization of automatic mask generators (AMG). **(a)** Original RGB image. **(b)** Previously annotated maize kernel instances highlighted by random colors.

Supplementary Figure S2 Annotation decisions on selected image crop from the leftmost ear in Figure 5. The colormap on the left side depicts the frequency a pixel has been assigned to a mask relative to the number of annotation rounds (*f_a, px_*) Only pixels assigned to a mask more than once are included. The right side shows the original RGB image.
