# Supplementary figures and images for: Orchestrating segment anything models to accelerate segmentation annotation on agricultural image datasets
Source: Front Artif Intell. 2026 Jan 22;8:1748468. doi: 10.3389/frai.2025.1748468 (PMC12872900; doi:10.3389/frai.2025.1748468)

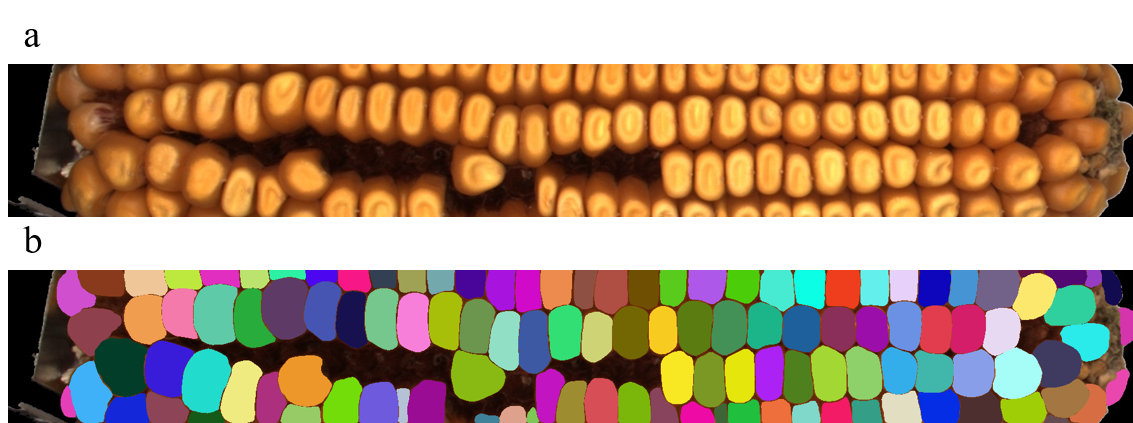

Supplement: Supplementary Figure S1 — Example of maize ear images used for hyperparameter optimization of automatic mask generators (AMG). (a) Original RGB image. (b) Previously annotated maize kernel instances highlighted by random colors. [file Image_1.TIF]

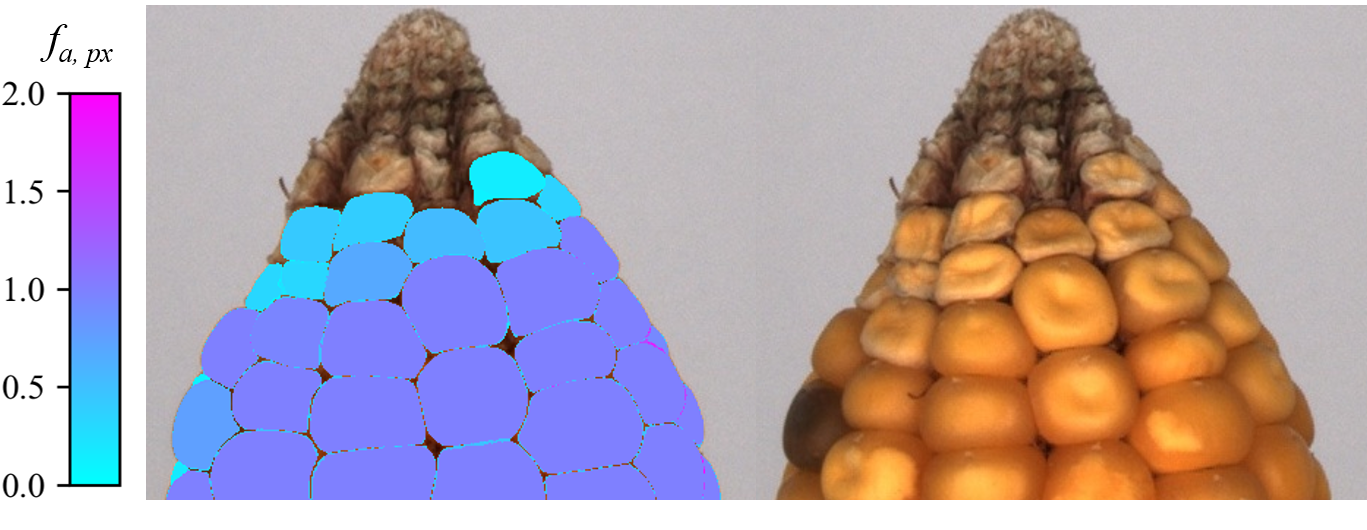

Supplement: Supplementary Figure S2 — Annotation decisions on selected image crop from the leftmost ear in Figure 5. The colormap on the left side depicts the frequency a pixel has been assigned to a mask relative to the number of annotation rounds (fa, px) Only pixels assigned to a mask more than once are included. The right side shows the original RGB image. [file Image_2.TIF]
